# Effects of Hydrogen Bonds between Ethoxylated Alcohols and Sodium Oleate on Collecting Performance in Flotation of Quartz

**DOI:** 10.3390/molecules28196945

**Published:** 2023-10-06

**Authors:** Na Zhang, Jiajia Li, Jue Kou, Chunbao Sun

**Affiliations:** School of Civil and Resource Engineering, University of Science and Technology Beijing, Beijing 100083, China; nazhang@ustb.edu.cn (N.Z.);

**Keywords:** hydrogen bond, molecular dynamics, ethoxylated alcohols, sodium oleate, quartz flotation

## Abstract

Hydrogen bonds play an important role in the interaction between surfactants. In this study, the effect of three different ethoxylated alcohols (OP-10, NP-10, AEO-9) on the collecting behavior of sodium oleate (NaOL) in the flotation of quartz was investigated. To explore the mechanism, the hydrogen bond between ethoxylated alcohols and NaOL was analyzed using molecular dynamics (MD) simulation. The results showed that ethoxylated alcohols promoted the collecting performance of NaOL and reduced the dosage of the activator CaO and the collector NaOL in the flotation of quartz. The Zeta potential measurement illustrated that ethoxylated alcohols promoted the adsorption of OL^−^ on the activated quartz surface and the degree of promotion was in the order of OP-10 > NP-10 > AEO-9. The MD simulation results showed that a hydrogen bond presented between ethoxylated alcohols and OL^−^. Due to the hydrogen bond between the ethoxylated alcohols and OL^−^, the attraction force between OL^−^ and the quartz surface increased with the addition of ethoxylated alcohols in the order of OP-10 > NP-10 > AEO-9 based on the MD simulation results. As the result, the addition of ethoxylated alcohols increased the adsorption density of OL^−^ on the activated quartz surface, which explained the promotion of the collecting performance of OL^−^ in the flotation of quartz.

## 1. Introduction

Froth flotation is a process that selectively separates materials based on whether they are water-repelling or have an affinity to water. Hydrophobic particles attach to bubble surfaces and become concentrated in froth, whereas hydrophilic particles stay in a pulp [1]. The surface behaviors of minerals and their interactions with other components (such as water, reagents and air) are the basis of flotation research [2]. The collector is the reagent used to modify the floatability of minerals, which adsorbs on mineral surface to make it hydrophobic and floatable [3]. To improve the performance of the collector, synergists are normally added during flotation [4,5], and the interactions between the synergist and collector molecules such as hydrogen bonds, electrostatic forces and van der Waals forces are key for the synergic effect [6].

Quartz is the predominant gangue mineral in iron ore, and reverse flotation is usually used to improve the concentrate grade in the cleaning stage [7]. In this process, sodium oleate (NaOL) is usually used as the collector to adsorb on the Ca^2+^-activated quartz surface, which makes the quartz surface hydrophobic for separation during flotation [8,9,10,11,12,13]. To improve the efficiency of NaOL as the collector, ethoxylated alcohols (R(OC_2_H_4_)_n_OH) have been employed as the synergists for NaOL during the flotation of quartz due to their advantages of low toxicity, rapid biodegradation and foamability [14,15,16].

**Figure 1:**
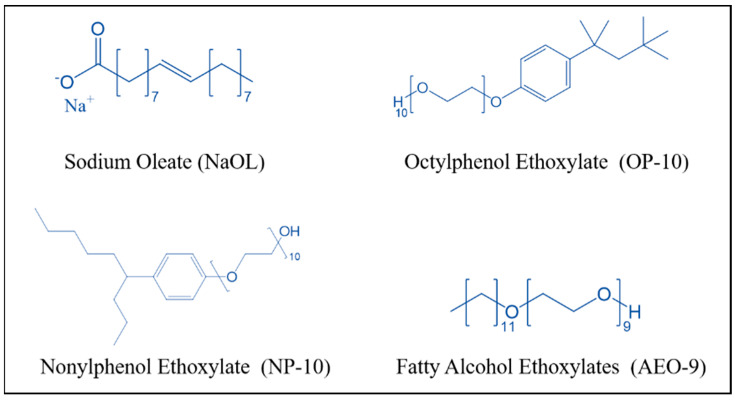
The chemical structure of NaOL, OP-10, NP-10 and AEO-9.

The noncovalent interactions between surfactants have been employed to obtain a variety of self-assembled structures such as micelles and vesicles [17,18]. It has been reported that there was synergism between oleate and a surfactant such as dodecane and D-phenylalanine through hydrogen bond interactions [19,20]. Ethoxylated alcohols are surfactants composed of elements C, H and O, whose polar head -CH_2_OH is the hydrogen bond donor and acceptor, and can form a hydrogen bond with oleate [21]. In previous research, the synergic effect of three ethoxylated alcohols, octylphenol ethoxylate (OP-10), nonylphenol ethoxylate (NP-10) and fatty ethoxylated alcohols (AEO-9), on the collecting performance of NaOL whose chemical structures are shown in Figure 1, was investigated, which certified that the addition of ethoxylated alcohols increased the surface activity and collecting performance of NaOL, resulting in the better flotation performance [14]. However, the previous study focused on the dilution effect of ethoxylated alcohol on the oleate based on regular solution theory (RST), while the hydrogen bond between them and its effect on the adsorption of oleate on the activated quartz surface needs to be further investigated.

In this study, the flotation behavior of quartz with the addition of ethoxylated alcohols (OP-10, NP-10, AEO-9) as synergists was investigated to determine the optimal dosage of reagents. The Zeta potential of quartz with the addition of ethoxylated alcohols was measured to investigate the effect of ethoxylated alcohols on the adsorption of NaOL on the quartz surface. A molecular dynamics (MD) simulation was adopted to investigate the hydrogen bonds between ethoxylated alcohols and OL^−^ as well as the adsorption energy between OL^−^ and the quartz surface with the addition of ethoxylated alcohols. Moreover, the effect of different ethoxylated alcohols on the adsorption density of OL^−^ on the quartz surface was also simulated.

## 2. Results

### 2.1. Flotation Behavior of Quartz with the Addition of Ethoxylated Alcohols

**Figure 2:**
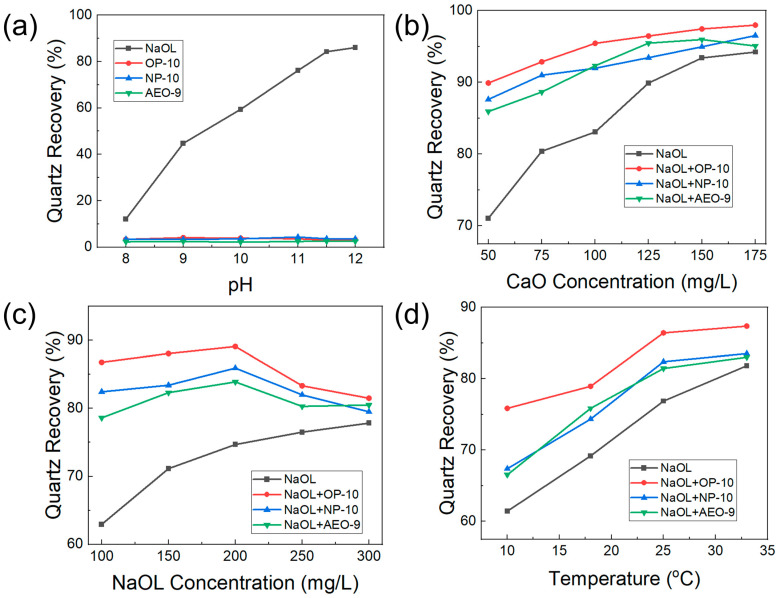
(**a**) The quartz recovery with 200 mg/L NaOL or 200 mg/L OP-10, NP-10, AEO-9 as the collector at different pH levels for flotation with 100 mg/L CaO. (**b**) The quartz recovery with different dosages of activator CaO in different collector systems with 200 mg/L NaOL without or with the addition of 5 wt% ethoxylated alcohols (OP-10, NP-10, AEO-9) at pH 11.5. (**c**) The change in quartz recovery with dosage of NaOL without or with 5 wt% ethoxylated alcohols (OP-10, NP-10, AEO-9) with 50 mg/L CaO at pH 11.5. (**d**) The change in quartz recovery with flotation temperature in different collector systems with 50 mg/L CaO, 150 mg/L NaOL without or with 7.5 mg/L ethoxylated alcohols (OP-10, NP-10, AEO-9) at pH 11.5.

The collecting behaviors of ethoxylated alcohols (OP-10, NP-10, AEO-9) were investigated with the addition of 100 mg/L CaO at different pH levels which were adjusted using NaOH solution. The collector was 200 mg/L NaOL or 200 mg/L OP-10, NP-10 or AEO-9. The results are shown in Figure 2a. The quartz recovery increased with pH when NaOL was the collector. The quartz recovery was 12.18% when the pH was 8, while it increased to 84.25% when the pH was 11.5, and then the increased rate of quartz recovery slowed down after pH 11.5. Therefore, pH 11.5 was selected as the optimal pH value for the flotation of quartz. In contrast, the quartz recovery was around 4% when OP-10, NP-10 or AEO-9 was the collector, which illustrated that the three ethoxylated alcohols (OP-10, NP-10, AEO-9) had no collecting effect on quartz. The 4% quartz recovery was due to the foaming ability of ethoxylated alcohols, which led to the entrainment of quartz during the flotation.

The optimal dosage of the activator CaO was investigated using 200 mg/L NaOL without or with 10 mg/L OP-10, NP-10, AEO-9 as the collector at pH 11.5. As shown in Figure 2b, the quartz recovery increased with the increase in CaO dosage in all collector systems. When NaOL was the collector, the quartz recovery increased from 71.07% to 94.22% with the dosage of CaO increasing from 50 mg/L to 175 mg/L. However, with the addition of ethoxylated alcohols, the quartz recovery increased under the same dosage of CaO in the order of OP-10 > NP-10 > AEO-9. With 50 mg/L CaO, the quartz recovery was 89.90%, 87.62% and 85.93% with the addition of OP-10, NP-10 and AEO-9, respectively. With the addition of OP-10, the quartz recovery with 50 mg/L CaO was higher than that with 125 mg/L CaO in the NaOL-only collector system. When the dosage of CaO was 175 mg/L, the quartz recovery was 97.97%, 96.52% and 95.06% with the addition of OP-10, NP-10 and AEO-9, respectively. After weighing the benefits and costs, 50 mg/L CaO was selected as the optimal dosage, under which the quartz recovery increased by 18.83% when NaOL + OP-10 was as the collector compared to NaOL only.

As the collector, the optimal dosage of NaOL was investigated with 50 mg/L CaO under pH 11.5, and the dosage of ethoxylated alcohols (OP-10, NP-10, AEO-9) was 5 wt% of NaOL. As shown in Figure 2c, without the addition of ethoxylated alcohols (OP-10, NP-10, AEO-9), the quartz recovery increased from 62.94% to 77.84% with the increased dosage of NaOL from 100 mg/L to 300 mg/L, all of which were below 80%. However, with the addition of ethoxylated alcohols (OP-10, NP-10, AEO-9), all quartz recovery was above 80% except that with NaOL (100 mg/L) + AEO-9 as the collector, which certified that ethoxylated alcohols enhanced the collecting performance of NaOL. With the increase in the dosage of NaOL, the quartz recovery increased and then decreased at the point of 200 mg/L NaOL with the addition of ethoxylated alcohols (OP-10, NP-10, AEO-9). The highest quartz recovery was 89.09%, 85.92% and 83.89% when the dosage of NaOL was 200 mg/L with the addition of OP-10, NP-10 and AEO-9, respectively. When the dosage of NaOL was 150 mg/L, the quartz recovery was 88.06%, 83.42% and 82.32% with the addition of OP-10, NP-10 and AEO-9, respectively, which was near to that with 200 mg/L NaOL. Therefore, from the economic and flotation efficiency points of view, the optimal dosage of NaOL was selected as 150 mg/L.

The performance of NaOL as the collector for quartz flotation was affected by the temperature of the pulp [11], so the optimal temperature for the flotation of quartz was investigated with 50 mg/L CaO and 150 mg/L NaOL without or with the addition of 7.5 mg/L ethoxylated alcohols (OP-10, NP-10, AEO-9) at pH 11.5. As shown in Figure 2d, when the temperature increased from 10 °C to 33 °C, the quartz recovery increased from 61.43% to 81.83% when NaOL was the collector. With the addition of ethoxylated alcohols (OP-10, NP-10, AEO-9), the quartz recovery increased in the order of OP-10 > NP-10 > AEO-9. With the increase in temperature, the quartz recovery also increased with the addition of ethoxylated alcohols (OP-10, NP-10, AEO-9). When the temperature was 10 °C, the quartz recovery with the addition of OP-10 was 75.86%, which was similar to that (76.87%) without the addition of ethoxylated alcohols at 25 °C. The quartz recovery was 86.41%, 82.38% and 81.44% at 25 °C with the addition of OP-10, NP-10 and AEO-9, respectively. As the temperature increases, the more Ca(OH)^+^ is produced, providing more sites for interaction with the collector, which increases the adsorption of NaOL on the quartz surface; therefore, the performance of NaOL increased with temperature [11]. The increased rate of quartz recovery slowed down with the addition of ethoxylated alcohols (OP-10, NP-10, AEO-9) when the temperature was over 25 °C, and 25 °C was the room temperature which needed no heating or cooling assistance, so 25 °C was set as the optimal temperature for quartz flotation.

Therefore, for the flotation of quartz, the optimal dosage of the reagent was 50 mg/L CaO, 150 mg/L NaOL and 7.5 mg/L ethoxylated alcohols (OP-10, NP-10, AEO-9) at 25 °C and pH 11.5. With the addition of ethoxylated alcohols (OP-10, NP-10, AEO-9), the performance of NaOL as the collector was enhanced, and OP-10 showed the best synergic effect.

### 2.2. Adsorption Behavior of OL^−^ on Quartz with the Participation of Ethoxylated Alcohols

**Figure 3:**
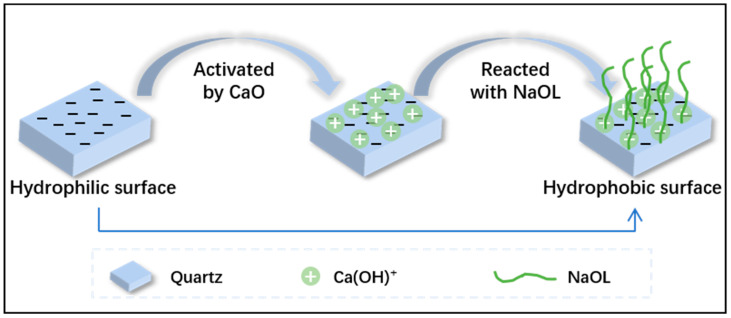
Scheme of the modification of quartz surface through activator CaO and collector NaOL.

The quartz surface was hydrophilic, which was not feasible for flotation, so it was modified to be hydrophobic and therefore floatable. The modification process is illustrated in Figure 3. The natural surface of quartz is negatively charged [22]. The activator CaO exists as Ca(OH)^+^ in water at pH 11.5, so Ca(OH)^+^ would adsorb on the quartz surface due to the electrostatic force [23,24]. Then, when NaOL was added to the flotation system, NaOL reacted with Ca(OH)^+^ on the quartz surface and generated -Si-O-Ca-OOCR bonds, so NaOL was adsorbed on the quartz surface and its hydrophobic tailing made the quartz surface hydrophobic [25]. The more OL^−^ adsorbed on the quartz surface, the more hydrophobic the quartz surface was, and the floatability of quartz was better.

**Figure 4:**
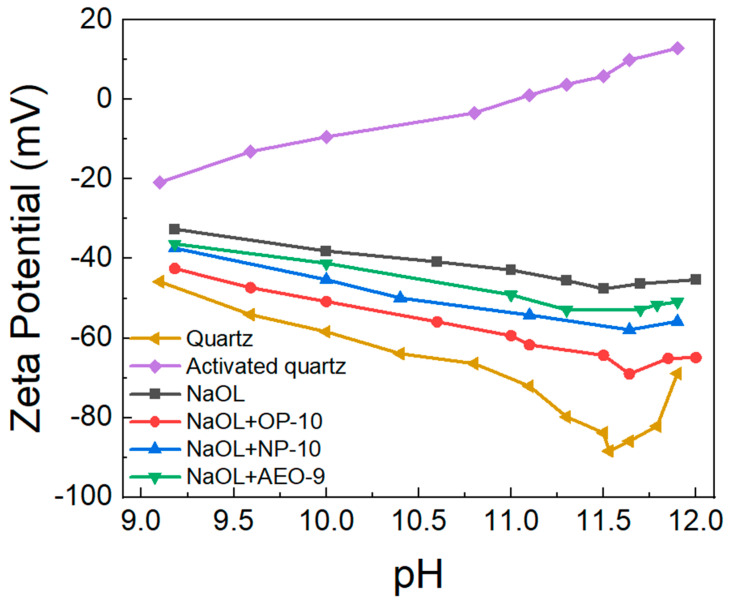
Zeta potential of quartz surface modified through different reagents at different pH levels.

The addition of ethoxylated alcohols (OP-10, NP-10, AEO-9) promoted the performance of NaOL in the flotation of quartz. To investigate the mechanism, the Zeta potential of the quartz surface after different modifications was detected. As shown in Figure 4, the quartz surface in deionized water without any modification was negatively charged; however, when it was activated through CaO, the negative surface charge was neutralized, and it became positively charged when the pH was over 11, which facilitated the adsorption of OL^−^. After the addition of NaOL, the activated quartz surface became negatively charged but the amount of charge was lower than the natural quartz surface. With the addition of ethoxylated alcohols (OP-10, NP-10, AEO-9), the amount of negative charge on the quartz surface was greater than that when only NaOL was added, which demonstrated that the addition of ethoxylated alcohols promoted the adsorption of NaOL on the quartz surface. The more negative the quartz surface was after the reaction with NaOL, the more OL^−^ adsorbed on the quartz surface, so the strength of the synergic effect of ethoxylated alcohols on the collecting ability of NaOL was in the order of OP-10 > NP-10 > AEO-9, which explained the results in Figure 2.

### 2.3. Hydrogen Bonds between Ethoxylated Alcohols and OL^−^ and Their Effect on Collecting Behavior of OL^−^

**Figure 5:**
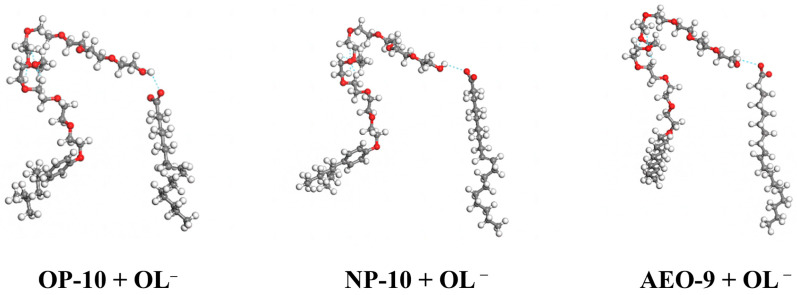
Hydrogen bonds generated between OL^−^ and OP-10/NP-10/AEO-9 (blue dash).

The hydrogen bonds generated between different ethoxylated alcohols and OL^−^ based on molecular dynamic simulation are shown in Figure 5. The element O in OL^−^ interacted with element H in the head -CH_2_OH of the ethoxylated alcohol molecule through a hydrogen bond O…H. Moreover, there were intramolecular hydrogen bonds in ethoxylated alcohol molecules, which made the ethoxylated alcohol molecule bent. From Figure 5, it can be learnt that the structure of the hydrogen bonds between OL^−^ and different ethoxylated alcohols was similar. Due to the hydrogen bonds between ethoxylated alcohols and OL^−^, there was steric hindrance between NaOL molecules in the micelles, so the electric free energy of micellization decreased [18].

The absolute values of hydrogen bond interactions between three different ethoxylated alcohols (OP-10, NP-10, AEO-9) and OL^−^ were calculated after MD simulation as shown in Table 1. Although the structures of hydrogen bonds between OL^−^ and different ethoxylated alcohols were similar, their strengths were different. The absolute value of the hydrogen bond between OL^−^ and OP-10, NP-10 and AEO-9 in vacuum was 21.94 kcal/mol, 10.23 kcal/mol and 7.58 kcal/mol, respectively, while their values in water was 4.57 kcal/mol, 2.30 kcal/mol and 1.46 kcal/mol, respectively. There was an obvious difference between the values in vacuum and water, which could be due to the effect of water on the charge of ethoxylated alcohols molecules and oleate [26]. A hydrogen bond is primarily the electrostatic force between H in ethoxylated alcohols and O in oleate [27]. The addition of water would affect the charge distribution in the molecules of ethoxylated alcohols and oleate; additionally, there would be hydrogen bonds between water and oleate or ethoxylated alcohol molecules, which both contributed to the decrease in hydrogen bond values in water. In both vacuum and water, the hydrogen bond interaction between OP-10 and OL^−^ was the largest. The generation of hydrogen bonds between ethoxylated alcohols and oleate decreased the repulsion between oleate caused by the negative atom O in micelles, which increased the activity of oleate [28,29].

The interaction energy change between OL^−^ and the activated quartz surface before and after the addition of ethoxylated alcohols (OP-10, NP-10, AEO-9) is shown in Table 2. The negative values indicate attractive interactions, and the greater the absolute value of the interaction energy is, the stronger the action intensity [30]. According to the data in Table 2, all the energy changes after the addition of ethoxylated alcohols were negative, so the addition of ethoxylated alcohols increased the adsorption energy between OL^−^ and activated quartz. Therefore, the ethoxylated alcohols promoted the adsorption of oleate on the activated quartz surface. The adsorption energy between OL^−^ and the activated quartz increased by 2264.9 kcal/mol, 1386.9 kcal/mol and 1234.5 kcal/mol with the addition of OP-10, NP-10 and AEO-9, respectively, so the addition of OP-10 promoted the adsorption of oleate the most.

**Figure 6:**
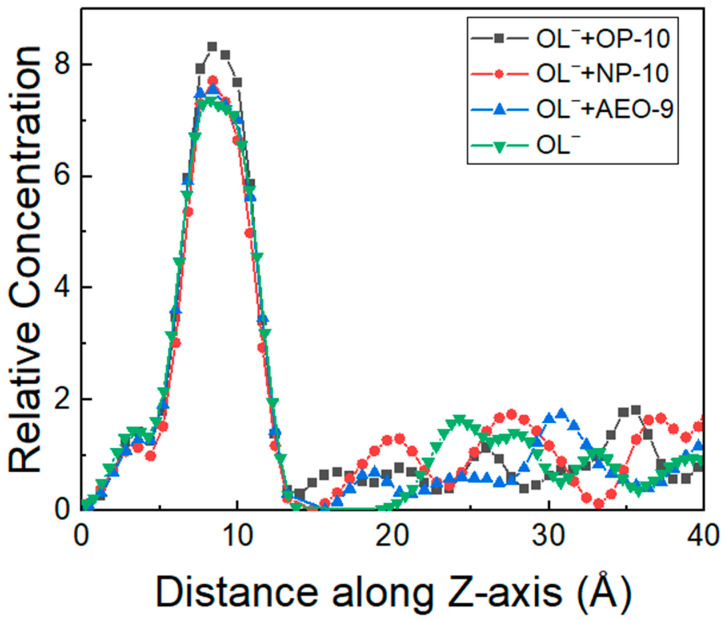
The relative concentration of OL^−^ on the activated quartz surface as a function of distance along the *Z*-axis without or with the addition of ethoxylated alcohols (OP-10/NP-10/AEO-9).

**Figure 7:**
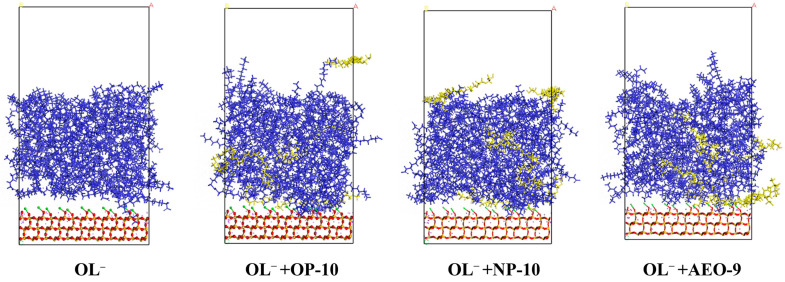
Molecular models of oleate on the activated quartz surface without or with the synergy of ethoxylated alcohols (OP-10/NP-10/AEO-9). OL^−^ is illustrated in blue and ethoxylated alcohols are shown in yellow.

As shown in Figure 6, the adsorption of oleate on quartz mainly occurred at the distance of 8.4 Å. Compared to the adsorption density with oleate only, the addition of ethoxylated alcohols increased the adsorption density of oleate in the order of OP-10 > NP-10 > AEO-9. The molecular model of oleate on the quartz surface is shown in Figure 7. When there were no ethoxylated alcohols added, there was separation between oleate and the quartz surface, and the density of oleate on the quartz surface was not as large as that with ethoxylated alcohols. With the addition of ethoxylated alcohols, the distance between oleate and the quartz surface decreased, and more oleate molecules were on the quartz surface because of the weakened repulsion between oleate, which was the result of hydrogen bonds between ethoxylated alcohols and oleate. The addition amount of ethoxylated alcohols was not large, but the synergic effect on the adsorption of OL^−^ on the quartz surface was obvious. Comparing the three different ethoxylated alcohols, when OP-10 was added, the number of oleate molecules on the quartz surface was the largest, and the distance between oleate and quartz was the smallest, which explained the results of flotation and Zeta potential well.

In summary, ethoxylated alcohols generated hydrogen bonds with oleate, which weakened the repulsion between oleate molecules and increased their activity. Therefore, ethoxylated alcohols increased the adsorption force between oleate and the activated quartz surface, and they increased the adsorption density of oleate on the quartz surface, which was consistent with the Zeta potential results. The adsorption of oleate made the quartz surface hydrophobic, which contributed to the flotation of quartz. Among those ethoxylated alcohols, the ionic hydrogen bond between OP-10 and oleate was the strongest, so the synergic effect of OP-10 on oleate in quartz flotation was the best.

## 3. Materials and Methods

### 3.1. Materials

The quartz was obtained from Yunnan Province, China. The fine quartz particles were obtained through crushing the quartz lumps using a hammer and then pulverizing them using a laboratory sample preparation pulverizer (Jiangxi Guangming Intelligent Technology Co., Ltd., Nanchang, China). Quartz particles with size of −38 μm were collected via sieving and were used in the flotation test. The quartz particles were soaked in HCl solution (4 mol/L) for 24 h to clean the surfaces, and the cleaning process was repeated twice. The cleaned quartz particles were rinsed with deionized water until the pH of the cleaning water was 7, which was followed by drying in an oven at 90 °C for 12 h. The purification of the quartz particles was 99.74%.

Deionized water whose conductivity was 0.054 μS/cm was used in this study. Sodium hydroxide (NaOH, Analytical Reagent) was purchased from Tianjin Fuchen Chemical Co., Ltd., Tianjin, China. Calcium oxide (CaO, ≥98%), sodium oleate (NaOL, ≥98%), nonylphenol ethoxylate (NP-10, ≥99.5%) and fatty ethoxylated alcohols (AEO-9, Chemically Pure) were purchased from Beijing Honghu Lianhe Huagong Chanpin Co., Ltd., Beijing, China. Octylphenol ethoxylate (OP-10, Chemically Pure) was purchased from Tianjin Bailunsi Biotechnology Co., Ltd. The chemical structures of NaOL, OP-10, NP-10 and AEO-9 are listed in Figure 1.

### 3.2. Flotation Test

A 50 mL hanging grooved XFGC_Ⅱ_ laboratory flotation machine (Jilin Exploration Machinery Plant, Jilin, China) was employed to perform flotation tests at room temperature (25 °C). First, 2.0 g quartz particles (−74 + 38 μm) and 30 mL deionized water were added to the flotation cell and stirred at 1992 r/min. The pH was adjusted to 11.5 through adding NaOH solution (75 mg/L) to the pulp. After the pulp was stirred for 3 min, 1.5 mg CaO (50 mg/L) was added to the pulp and was stirred for another 3 min to activate the quartz particles. After that, the collector, NaOL (150 mg/L) with 0.3 mg (7.5 mg/L) ethoxylated alcohols (OP-10, NP-10, AEO-9), was added to the pulp and was stirred for 3 min. The froths were collected at the air flow rate of 1 L/min for 4 min. To make sure the froths could be collected, a moderate amount of deionized water was added during the collection of froths. The collected froth and the tailings were filtrated and dried separately, and the quartz recovery was measured using the mass of the froths and tailings.

A variable temperature test was performed through controlling the temperature of the pulp. The deionized water in the flotation cell was set to the desired temperature using a thermometer, and the temperature was controlled through adding hot water when minerals and reagents were added to the flotation cell. During the froth collection process, the temperature was also controlled at the desired temperature.

### 3.3. Zeta Potential Test

The Zeta potential of the quartz surface in different conditions was measured with a Zetasizer Nano ZS90 (Malvern Instrument Ltd., Malvern, UK). In total, 50 mL deionized water and 0.2 g quartz particles, which were ground to −5 μm using an onyx mortar, were added to a 100 mL beaker, and then the pH of the pulp was adjusted through adding NaOH (300 mg/L), and then CaO (50 mg/L), NaOL (150 mg/L) or NaOL (150 mg/L) + OP-10, NP-10, AEO-9 (7.5 mg/L) were added in order. The modified quartz particles were filtered through the filter paper with a particle retention of 25 μm after stirring for 30 min. The pH value and Zeta potential of the filtrate were measured. The Zeta potential of NaOL (150 mg/L) or NaOL (150 mg/L) + OP-10, NP-10, AEO-9 (7.5 mg/L) in deionized water was also tested for the EDLVO calculation. Every Zeta potential test was repeated five times.

### 3.4. Molecular Dynamic (MD) Simulation

The MD simulation was performed using the Materials Studio (MS) 2020 software package (BIOVIA, Waltham, MA, USA). The model of quartz was chosen from the built-in database of MS and then optimized with the CASTEP module. The dominant cleavage plane for calculation was set as the 101 quartz surface [11,31]. After that, the Ca^2+^-activated quartz surface was constructed through introducing calcium species and subsequently treated with further optimization using the CASTEP module.

The interaction model with a size of 44 Å × 40 Å × 80 Å (a × b × c) was composed of three layers [32]. The quartz slab was the bottom layer, and the sodium oleate solution case was the top layer. The Amorphous Cell module was used to build the box with 6 ethoxylated alcohol molecules and 120 NaOL molecules. The Forcite Module was used to optimize the molecular structure with COMPASS II as the force field [30]. The relative concentration of OL(−) depicted was a function of its center-of-mass (COM) to the quartz surface. During the geometric optimization process, the convergence threshold for maximum energy change, maximum force convergence threshold and maximum displacement convergence threshold were 0.001 kcal/mol, 0.5 kcal/mol/Å and 0.015 Å, respectively. In order to obtain a more optimized structure, a 200 ps dynamic simulation was conducted under the NVT ensemble with a step size of 0.5 fs. In the process of MD, electrostatic potential was calculated using Ewald, and the periodic boundary conditions were true for all three directions, while van der Waals forces were calculated using Atom base. The precise Nose–Hoover temperature control mode (298.15 K) and Berendsen pressure control mode were used in the dynamic process. The density of OL^−^ along the *z*-axis was obtained through selecting OL^−^ as set in Forcite analysis. The interaction energy between quartz and oleate was calculated using the following equations [11]:

E_int1_ = E_sum1_ − E_q_ − E_o+EA_

E_int2_ = E_sum2_ − E_q_ − E_o_

ΔE_int_ = E_int1_ − E_int2_

where E_sum1_ represents the total energy of quartz, ethoxylated alcohols (EA) and oleate in the system. E_sum2_ represents the total energy of quartz and oleate in the system. While E_q_, E_o_ and E_o+EA_ represent the energy of quartz, oleate and the sum of oleate and AE, respectively. E_int1_ is the interaction energy o of oleate on the quartz surface when ethoxylated alcohols (EA) were added. E_int2_ is the interaction energy of quartz and oleate. ΔE_int_ represents the difference in the change in action energy before and after the addition of ethoxylated alcohols.

To compare the hydrogen bonds between ethoxylated alcohols and oleate in the vacuum and water, another amorphous cell module was used to build the box with 6 ethoxylated alcohol molecules, 30 sodium oleate molecules and 2000 water molecules. Intermolecular hydrogen bond diagrams for ethoxylated alcohols and OL^−^ were obtained through the function of “calculate hydrogen bonds” in Materials Studio software. The maximum donor (D)–acceptor (A) distance d (D…A) was set as 0.25 nm. Hydrogen bonding energy was obtained through scripts as follows:

#!perl

use strict;

use Getopt::Long;

use MaterialsScript qw(:all);

my $doc = $Documents{“A.xsd”};

print “Hydro_Energy = %0.3f”, $doc->HydrogenBondEnergy

## 4. Conclusions

The effects of hydrogen bonds between ethoxylated alcohols and sodium oleate on the collecting performance in the flotation of quartz were investigated using the Zeta potential and MD simulation. The main conclusions are as follows:

• The ethoxylated alcohols (OP-10, NP-10, AEO-9) had no collecting ability, but they could promote the flotation of quartz when sodium oleate was the collector. The dosage of the reagent including the activator CaO and the collector NaOL could be decreased with the addition of ethoxylated alcohols (OP-10, NP-10, AEO-9) to achieve the same flotation index. The promotion effect was in the order of OP-10 > NP-10 > AEO-9. The optimal dosage of reagent was 50 mg/L CaO, 150 mg/L NaOL and 7.5 mg/L ethoxylated alcohols (OP-10, NP-10, AEO-9), and the optimal temperature and pH were 25 °C and 11.5, respectively.
• Based on the change in the Zeta potential of the quartz surface after modification, ethoxylated alcohols (OP-10, NP-10, AEO-9) promoted the adsorption of oleate on the quartz surface in the order of OP-10 > NP-10 > AEO-9.
• Based on the MD simulation results, it was shown that there were hydrogen bonds between ethoxylated alcohols and oleate molecules, which decreased the repulsion between oleate in micelles, increased the activity of oleate and increased the attraction force between oleate and the activated quartz surface. As a result, the adsorption density of oleate increased with the synergy of ethoxylated alcohols in the order of OP-10 > NP-10 > AEO-9, which explained the results of the Zeta potential and flotation well.

## Figures and Tables

**Table 1:** The absolute value of hydrogen bond energy between different ethoxylated alcohols and OL^−^ in vacuum or water.

Alcohol Ethoxylate	Hydrogen Bond Energy in Vacuum (kcal/mol)	Hydrogen Bond Energy in Water (kcal/mol)
OP-10	21.94	4.57
NP-10	10.23	2.30
AEO-9	7.58	1.46

**Table 2:** The interaction energy between OL^−^ and the activated quartz surface with the synergy of OP-10, NP-10 and AEO-9.

Alcohol Ethoxylate (AE)	Energy Change after Addition of AE (kcal/mol)
OP-10	−2264.9
NP-10	−1386.9
AEO-9	−1234.5

## Data Availability

Not applicable.

## References

[1] Crawford C.B., Quinn B., Crawford C.B., Quinn B. (2017). 9—Microplastic Separation Techniques. Microplastic Pollutants.

[2] Cui W., Chen J. (2021). Insight into mineral flotation fundamentals through the DFT method. Int. J. Min. Sci. Technol..

[3] Feng Q., Yang W., Wen S., Wang H., Zhao W., Han G. (2022). Flotation of copper oxide minerals: A review. Int. J. Min. Sci. Technol..

[4] Aarab I., Derqaoui M., Abidi A., Yaacoubi A., El Amari K., Etahiri A., Baçaoui A. (2020). Direct flotation of low-grade Moroccan phosphate ores: A preliminary micro-flotation study to develop new beneficiation routes. Arab. J. Geosci..

[5] Ren Z., Shen Y., Gao H., Chen H., Liu C., Chen Z. (2021). Comparison of Sodium Oleate and Sodium Petroleum Sulfonate for Low-Temperature Flotation of Fluorite and the Collecting Mechanisms. Min. Metall. Explor..

[6] Qiu H., Wu B., Deng J., Sun X., Hu M., Cai J., Zheng C. (2022). The effect of collectors on froth stability of frother: Atomic-scale study by experiments and molecular dynamics simulations. J. Mol. Liq..

[7] Xiong D., Lu L., Holmes R.J., Lu L. (2015). 9—Developments in the physical separation of iron ore: Magnetic separation. Iron Ore.

[8] Ozkan A., Ucbeyiay H., Duzyol S. (2009). Comparison of stages in oil agglomeration process of quartz with sodium oleate in the presence of Ca(II) and Mg(II) ions. J. Colloid Interface Sci..

[9] Hao H., Li L., Yuan Z., Liu J. (2018). Molecular arrangement of starch, Ca^2+^ and oleate ions in the siderite-hematite-quartz flotation system. J. Mol. Liq..

[10] Wang Y., Ahmed Khoso S., Luo X., Tian M. (2019). Understanding the depression mechanism of citric acid in sodium oleate flotation of Ca^2+^-activated quartz: Experimental and DFT study. Miner. Eng..

[11] Cao S., Yin W., Yang B., Zhu Z., Sun H., Sheng Q., Chen K. (2022). Insights into the influence of temperature on the adsorption behavior of sodium oleate and its response to flotation of quartz. Int. J. Min. Sci. Technol..

[12] Liu A., Fan P.-P., Qiao X.-X., Li Z.-H., Wang H.-F., Fan M.-Q. (2020). Synergistic effect of mixed DDA/surfactants collectors on flotation of quartz. Miner. Eng..

[13] Cao Q., Cheng J., Wen S., Li C., Bai S., Liu D. (2015). A mixed collector system for phosphate flotation. Miner. Eng..

[14] Zhang N., Li J., Kou J., Sun C. (2023). Synergy Effect between Sodium Oleate and Ethoxylated alcohols on the Reverse Flotation of Quartz. Minerals.

[15] Espeso M.B., Corada-Fernández C., García-Delgado M., Candela L., González-Mazo E., Lara-Martín P.A., Jiménez-Martínez J. (2021). Structural control of the non-ionic surfactant ethoxylated alcohols (AEOs) on transport in natural soils. Environ. Pollut..

[16] Li Y., Zhou J., Zhang Y., Liang H., Sun J., Liu Y., D’Errico G., Sun Y., Di Serio M. (2021). Synthesis and Properties of Primary Ethoxylated alcohols Using Different Catalytic Systems. ACS Omega.

[17] Zhou C., Cheng X., Zhao O., Liu S., Liu C., Wang J., Huang J. (2014). The evolution of self-assemblies in the mixed system of oleic acid–diethylenetriamine based on the transformation of electrostatic interactions and hydrogen bonds. Soft Matter.

[18] Ghosh S., Ray A., Pramanik N. (2020). Self-assembly of surfactants: An overview on general aspects of amphiphiles. Biophys. Chem..

[19] An M., Liao Y., Cao Y., Hao X., Ma L. (2021). Improving Low Rank Coal Flotation Using a Mixture of Oleic Acid and Dodecane as Collector: A New Perspective on Synergetic Effect. Processes.

[20] Li H., Chai W., Cao Y., Wu Y., Yang S. (2021). Synergistic collection mechanism of D-phenylalanine and sodium oleate in flotation of diaspore from kaolinite. Appl. Surf. Sci..

[21] Ayoub A.T., Tuszynski J., Klobukowski M. (2014). Estimating hydrogen bond energies: Comparison of methods. Theor. Chem. Acc..

[22] Luo X., Wang Y., Wen S., Ma M., Sun C., Yin W., Ma Y. (2016). Effect of carbonate minerals on quartz flotation behavior under conditions of reverse anionic flotation of iron ores. Int. J. Miner. Process..

[23] Kou J., Xu S., Sun T., Sun C., Guo Y., Wang C. (2016). A study of sodium oleate adsorption on Ca^2+^ activated quartz surface using quartz crystal microbalance with dissipation. Int. J. Miner. Process..

[24] Hou Y., Sobhy A. (2021). New Insights on Sodium Oleate Adsorption on Quartz for Iron Direct Flotation under Weak-Acidic Condition. Tenside Surfactants Deterg..

[25] Cao Z., Zhang Y., Cao Y. (2013). Reverse Flotation of Quartz From Magnetite Ore with Modified Sodium Oleate. Miner. Process. Extr. Metall. Rev..

[26] Ben-Tal N., Sitkoff D., Topol I.A., Yang A.-S., Burt S.K., Honig B. (1997). Free Energy of Amide Hydrogen Bond Formation in Vacuum, in Water, and in Liquid Alkane Solution. J. Phys. Chem. B.

[27] Sweetman A.M., Jarvis S.P., Sang H., Lekkas I., Rahe P., Wang Y., Wang J., Champness N.R., Kantorovich L., Moriarty P. (2014). Mapping the force field of a hydrogen-bonded assembly. Nat. Commun..

[28] Zhou Q., Rosen M.J. (2003). Molecular Interactions of Surfactants in Mixed Monolayers at the Air/Aqueous Solution Interface and in Mixed Micelles in Aqueous Media: The Regular Solution Approach. Langmuir.

[29] Holland P.M., Rubingh D.N. (1992). Mixed surfactant systems: An overview. Mixed Surfactant Systems.

[30] Sun H. (1998). Compass: An ab initio force-field optimized for condensed-phase applications—Overview with details on alkane and benzene compounds. J. Phys. Chem. B.

[31] Wang X., Liu W., Liu W., Shen Y., Duan H., Qiu J., Gu X. (2021). Understanding adsorption of amine surfactants on the solvated quartz (101) surface by a jointed Dreiding-ClayFF force field. Appl. Surf. Sci..

[32] Luo X., Qi L., Wen S., Wang Y., Lai H., Lin Q., Zhou Y., Wu X., Song Z. (2021). Adsorption configuration of dodecylamine at gas–liquid interface and its relationship with foam stability: MD simulation and ToF-SIMS investigation. Miner. Eng..

